# Pancancer Analysis of the Prognostic and Immunotherapeutic Value of Progestin and AdipoQ Receptor 4

**DOI:** 10.1155/2022/2528164

**Published:** 2022-12-17

**Authors:** Ziyue Yang, Yuanyuan Zhu, Zhenfen Li, Zhangya Pu, Ying Lin, Ying Deng, Ning Li, Fang Peng

**Affiliations:** ^1^Department of Blood Transfusion, Xiangya Hospital, Central South University, 87 Xiangya Road, Changsha, Hunan Province 410008, China; ^2^NHC Key Laboratory of Cancer Proteomics, Xiangya Hospital, Central South University, 87 Xiangya Road, Changsha, Hunan Province 410008, China; ^3^Department of Infectious Diseases and Hunan Key Laboratory of Viral Hepatitis, Xiangya Hospital, Central South University, 87 Xiangya Road, Changsha, Hunan Province 410008, China; ^4^Department of Nuclear Medicine, Xiangya Hospital, Central South University, Changsha, China; ^5^People's Hospital of Ningxiang, Changsha, Hunan Province 410600, China

## Abstract

AdipoQ receptor 4 (PAQR4) belongs to the family of progestin and AdipoQ receptors. PAQR4 plays an oncogenic role in lung and breast cancer. However, systematic pancancer analyses of PAQR4 have not been performed. The purpose was to investigate the prognostic and immunological significance of PAQR4 across 31 tumor types. Data were obtained from the following sources: TCGA, GEO, UALCAN, TIMER, GEPIA2, KM plotter, and TISIDB databases. The results proved that PAQR4 expression was significantly elevatory in most cancer types. We then explored the utility of PAQR4 as a prognostic indicator across all cancers. Using Cox proportional risk regression models, it has been demonstrated that PAQR4 is an independent risk factor in. High PAQR4 expression was not associated with other prognostic indicators, including overall survival, disease-free interval, disease-specific survival, and progression-free period. Subsequently, we explored the immunological value of PAQR4 and found that PAQR4 expression significantly correlated with tumor mutational burden, microsatellite instability, neoantigen, and immune checkpoint genes in tumors. It also significantly negatively correlated with most tumors' ESTIMATE scores, indicating that PAQR4 can influence the cellular composition of the tumor microenvironment. Our findings suggest the immunotherapeutic potential of PAQR4 in tumors. Finally, we explored the role of PAQR4 in tumor drug resistance and found that PAQR4 expression affected the sensitivity to multiple chemotherapeutic agents. A significant role for PAQR4 in tumor immunity is evident in these studies, as well as its potential role in cancer diagnosis, prognosis, and treatment precision.

## 1. Introduction

Malignant tumors have gradually become the most significant factor affecting human life [1]. Due to the insidious nature and the lack of clinical symptoms in early stage, it is difficult to achieve early diagnosis and treatment during the optimal window of tumor treatment [2]. In recent years, tremendous advances have been made in the treatment of tumors, with chemotherapy and radiation therapy significantly improving the five-year survival rate of patients. However, we still face many problems. Chemotherapy and radiation therapy bring severe side effects, which prevent patients from tolerating the intensity of treatment. This leads to poor compliance, which seriously affects the efficacy of the treatment. Immunotherapy represented by PD1 and PDL1 has entered clinical practice, particularly immune checkpoint blockade therapy, which has achieved outstanding progress [3]. Due to the heterogeneity of malignant tumors, more immunotherapeutic targets need to be explored, and the blueprint for tumor immunotherapy needs to be expanded [4].

The next-generation sequencing and single-cell transcriptome technologies allow us to explore the complex gene regulatory network of tumors at the transcriptional level and even at the single-cell level. The application of sequencing technology has improved clinical diagnosis and provided many therapeutic options. Meanwhile, as on-limits databases such as the Human Cancer Genome Atlas (TCGA) and GEO continue to improve, transcriptomic data could be linked to different clinical modules. Individual genes can be comprehensively analyzed for their broad role in different tumors [5]. Gene expression can be combined with a variety of factors such as patient survival, treatment, and drug sensitivity. By analyzing genes across cancer types, we can explore and evaluate their potential for clinical prognosis and targeted therapy [6].

As an AdipoQ receptor, PAQR4 belongs to the family of AdipoQ receptors [7]. This family encodes functional receptors with broad ligand specificity and is involved in a variety of biomodulatory processes, including hormone secretion and tumor progression [8]. The PAQR4 protein consists of seven transmembrane helices and is anchored within the Golgi membrane [9]. A key role for PAQR4 in tumor development has been demonstrated. An antagonistic crosstalk between PAQR4 and SKP2 regulates the homeostatic level of CDK4, which promotes cell proliferation and tumor regulation [10]. One study found that PAQR4 could activate the PI3K/AKT pathway promoting hepatocellular carcinoma development [11]. PAQR4 is also involved in the malignant ability of NSCLC through the CDK4-pRB-E2F1 pathway and can promote chemotherapy resistance in NSCLC by inhibiting Nrf2 protein degradation [12]. Research has shown that PAQR4 is highly expressed in breast carcinoma and activates antiapoptotic ceramidase to promote tumor proliferation [9]. Previous studies on PAQR4 have shown that it promotes tumor metastasis, which directly affects patient prognosis. Consequently, we infer that PAQR4 is a novel oncogene involved in multiple pathways promoting cancer development and is a potential prognostic and therapeutic pancancer biomarker.

However, current studies of PAQR4 in tumors have been limited to a specific type of cancer. There has been no study of the association between PAQR4 and pancancer. And, there have also been no immunological studies of PAQR4, which is necessary for further immunotherapy. We conducted a comprehensive and systematic study of PAQR4 using bioinformatic methods, including expression profiles, prognostic significance, and immunological values of PAQR4. Furthermore, PAQR4 expression was correlated with drug sensitivity. Our analysis across pancancer provides new perspective into the study of PAQR4 in cancer, uncovers immunological and epigenetic mechanisms, and provides new strategies for the early diagnosis and immunotherapy of tumors.

## 2. Materials and Methods

### 2.1. TIMER, GEPIA2, and CLLE

TIMER (https://cistrome.shinyapps.io/timer/) is a webpage based on the TCGA, which calculates the abundance of immune cell infiltration in different tumor tissues and gene expression differences between tumor and normal tissues [13]. In this work, the “DiffExp module” was used to explore differential PAQR4 expression between tumor and tumor-adjacent tissues across different tumor types. The “Gene plate” was used to explore the correlation between PAQR4 expression and immune infiltration level.

GEPIA2 (http://gepia2.cancer-pku.cn/#index) combines the TCGA and GTEx databases for differential analysis [14] and uses log2(TPM + 1) for logarithmic scaling. We considered *P* < 0.01 to be statistically significant. The relationship between PAQR4 and overall survival (OS) and disease-free survival (DFS) was calculated on GEPIA2, and PAQR4 expression was cut off at the median.

Based on the CCLE dataset, the pancancer cell line expression was calculated (https://portals.broadinstitute.org/ccle/about) [15], and the expression matrix was constructed using the R studio (v4.2.0) and package ggplot2.

### 2.2. PrognoScan and KM Plotter

The PrognoScan (http://dna00.bio.kyutech.ac.jp/PrognoScan/index.html) website is used to explore the relationship between gene expression and patient clinical prognoses, including OS and DFS.

The KM plotter [16] was used to analyze the relationship between PAQR4 and OS across pancancer. The log-rank test was used for comparative analysis of survival in different groups, and the significance level was set at *P* < 0.05.

### 2.3. TISIDB

In the TISIDB (http://cis.hku.hk/TISIDB/), multiple data types are integrated to predict tumor-immune interactions [17]. The correlation of PAQR4 with the immune subtypes of most cancers was analyzed using the TISIDB database. Pearson correlation was used, and *P* value of 0.05 was set as the statistical significance level.

### 2.4. Sangerbox

Based on the TCGA and GTEx databases, Sangerbox is an online analysis tool. The tool downloads data from the UCSC (https://xenabrowser.net/) and analyzes them online. A Sangerbox analysis was performed to assess the correlation between PAQR4 expression and immune checkpoint genes, tumor mutation burden, microsatellite instability, neoantigens, and ESTIMATE in tumors.

To further confirm PAQR4's prognostic value, Cox proportional hazard risk regression models were employed, and the association of PAQR4 expression with OS, disease-specific survival (DSS), disease-free interval (DFI), and progression-free period (PFI) in different malignant tumor patients was observed using the Sangerbox. The expression values were transformed by log2(x + 0.001), and correlation analysis was performed using the Pearson correlation coefficient.

### 2.5. UALCAN

UALCAN (http://ualcan.path.uab.edu) is a site that provides comprehensive and interactive information. It enables online analysis of different types of tumor data based on the TCGA [18]. In our research, UALCAN was used to analyze differences in PAQR4 promoter methylation levels between tumor and normal tissues. The promoter methylation levels were expressed by beta values, and the Student *t*-test was used for statistically significant comparisons.

### 2.6. cBioPortal

cBioPortal (https://www.cbioportal.org) is a platform for exploring multidimensional cancer genomic data based on the TCGA. It was used to analyze mutations in the PAQR4 gene in different cancers, including gene mutation summary and pan-oncogene mutation mapping.

### 2.7. GSCA

GSCA (http://bioinfo.life.hust.edu.cn/GSCA/#/) integrates the drug sensitivity and transcriptomics data of cancer cell lines in GDSC (genomics of drug sensitivity in cancer) and CTRP (the cancer therapeutics' response portal) [19]. In this study, GSCA was used to analyze the frequency of harmful mutations in PAQR4 in kinds of cancer types and to analyze the correlation between PAQR4 expression and copy number variations (CNV). A Spearman correlation coefficient was used to analyze the correlation between PAQR4 expression level and drug sensitivity (including various chemotherapy drugs).

### 2.8. CellMiner

The CellMiner (https://discover.nci.nih.gov/cellminer/home.do) is a tool for integrating and studying pharmacological data of 60 tumor cell types [20]. Processed RNA sequencing and Developmental Therapeutics Program NCI-60 datasets were downloaded from CellMiner. The R Studio software (v4.1.2) was used for statistical analysis and mapping. The analysis focuses on FDA-approved or clinically tested drugs. Then, the correlation of PAQR4 with drug IC50 was analyzed using the “impute,” “limma,” R packages.

## 3. Results

### 3.1. PAQR4 Expression Is Higher in Tumors than in Normal Tissues

**Figure 1:**
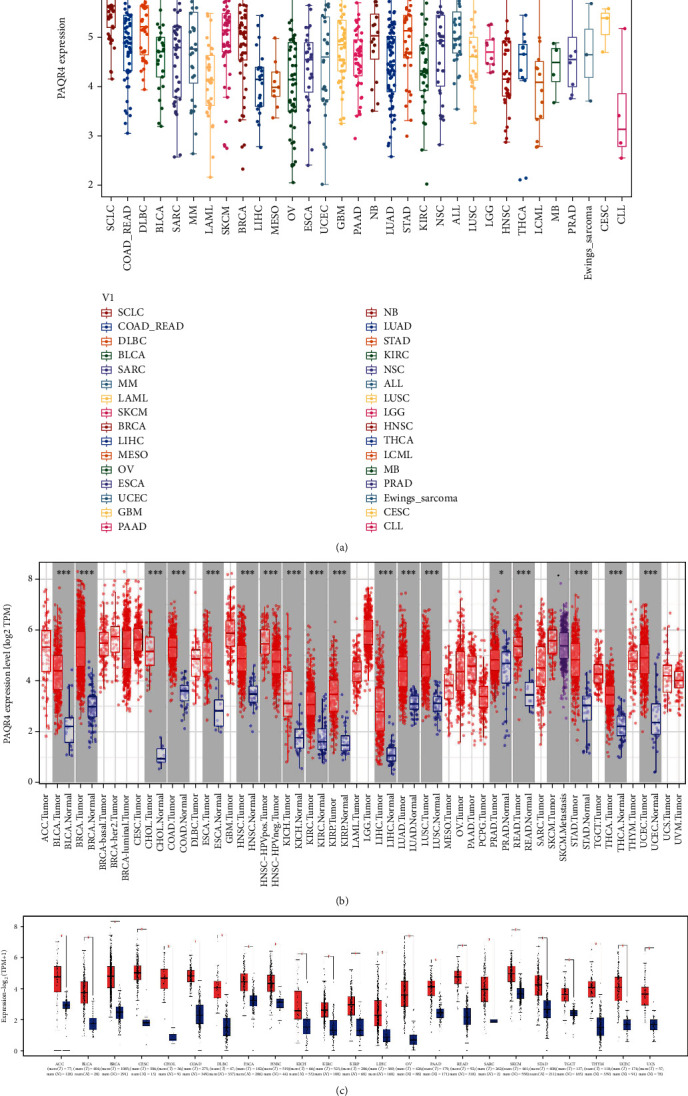
PAQR4 expression levels in human cancers. (a) PAQR4 expression in different cancer cell lines. It was exceptionally high in SCLC cell lines and lower in MESO cell lines. (b) PAQR4 expression differences in tumors and normal tissues from the TCGA database analyzed by the TIMER database, and PAQR4 is significantly higher in 17 tumors. (^∗^*P* < 0.05, ^∗∗^*P* < 0.01, and ^∗∗∗^*P* < 0.001). (c) PAQR4 expression differences in tumor and normal tissues in the GEPIA2 database, red is for tumor tissue and blue is for normal tissue. (^∗^*P* < 0.05).

First, we investigated PAQR4 expression levels in different tumor cell lines (Figure 1(a)). PAQR4 was differentially expressed in tumor cell lines and was exceptionally high in SCLC (small cell lung cancer) and low in MESO (mesothelioma) cell lines.

Furthermore, we explored the differences in PAQR4 expression between tumor and normal tissues. GEPIA2 website differential expression analysis showed that PAQR4 is significantly overexpressed in 23 tumor types (Figure 1(c)), and the full names and abbreviations of the 23 tumor types are shown in Supplementary Table 1. To further detect the pancancer PAQR4 expression level, we analyzed the TCGA RNA sequencing data using the TIMER website and found high PAQR4 expression in 17 tumor types (Figure 1(b)). These data demonstrate that PAQR4 is significantly highly expressed in most tumors.

### 3.2. The Pancancer Prognostic Value of PAQR4

**Figure 2:**
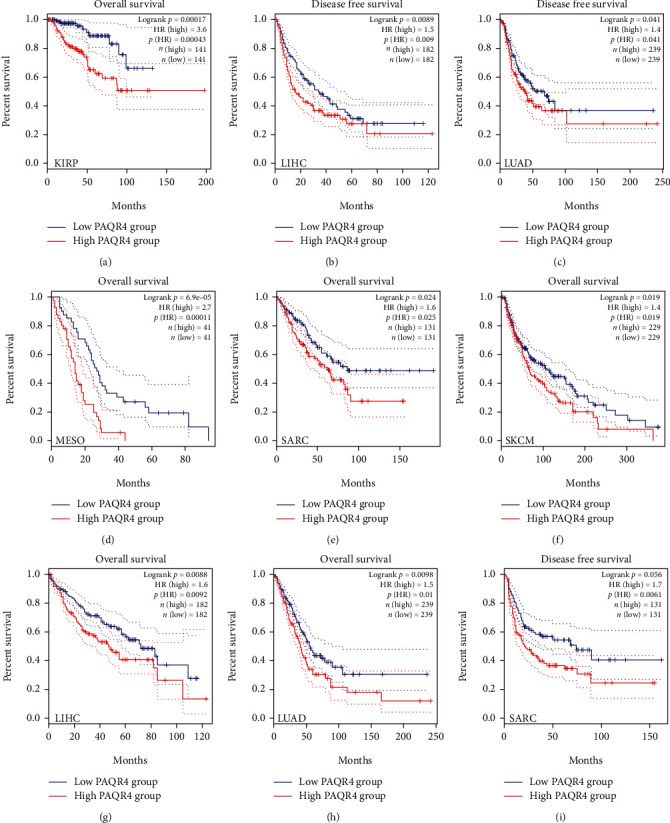
(a–i) Kaplan-Meier survival curve of human cancers with high and low PAQR4 expression group analyzed by the GEPAI2 database.

**Figure 3:**
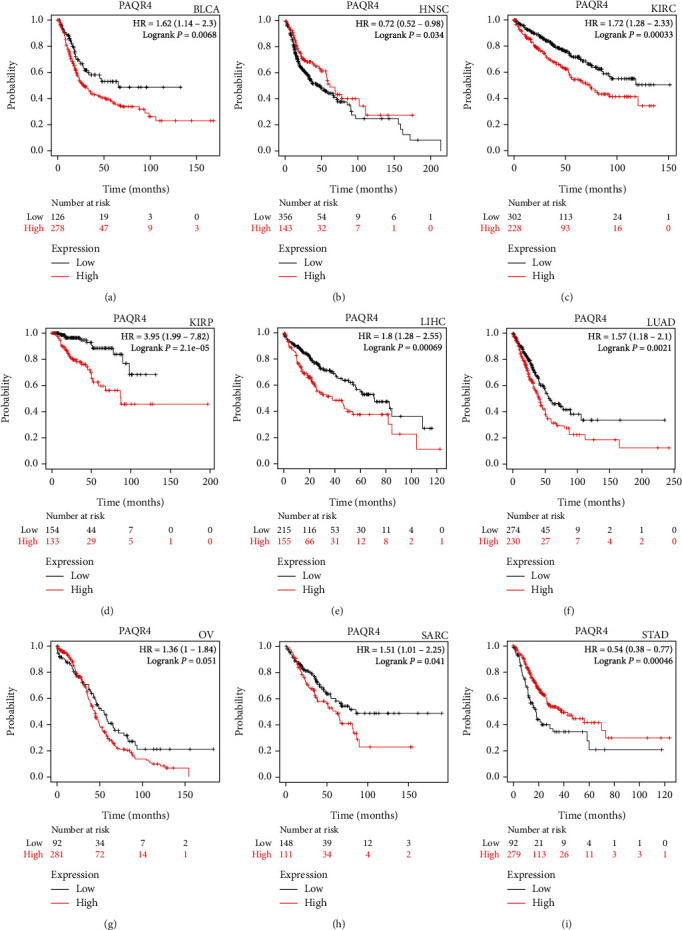
Survival analyses of the PAQR4 in human cancers in the Kaplan-Meier plotter database. OS was significantly worse in the PAQR4 high expression group: (a) BLCA, (c) KIRC, (d) KIRP, (e) LIHC, (f) LUAD, (g) OV, (h) and SARC. In (b) HNSC and (i) STAD, PAQR4 is a prognosis-affecting protective factor.

To explore the pancancer prognostic value of PAQR4, a series of analyses were performed. Using GEPIA2, as a risk factor, PAQR4 significantly affected OS and DFS in KIRP, LIHC, LUAD, MESO, SARC, and SKCM (Figure 2), and the PAQR4 high expression group showed worse OS, DFS, and shorter median survival time. Using the Kaplan-Meier plotter, similar outcomes were found. OS was significantly worse in the PAQR4 high expression group: BLCA, KIRC, KIRP, LIHC, LUAD (lung adenocarcinoma), OV, and SARC, and PAQR4 was a risk factor affecting the prognosis of these tumors. However, in HNSC and STAD, OS for the PAQR4 high expression group was longer than that for the low expression group, indicating that PAQR4 is a prognosis-affecting protective factor (Figure 3).

**Figure 4:**
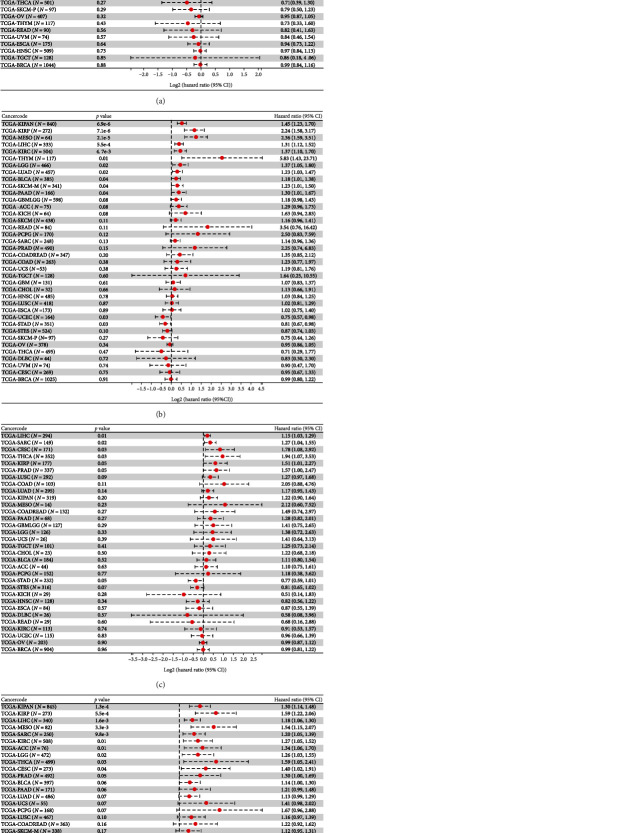
Correlation analysis of PAQR4 expression with (a) OS, (b) DSS, (c) PFI, and (d) DFI. Data are shown as forest plots.

We then used the Sangerbox online tool, which uses the R package surv's Coxph function, to build Cox proportional hazard risk regression models. In 44 tumors, we identified a linkage between PAQR4 expression and OS, DSS, DFI, and PFI. Poor OS occurred with high PAQR4 expression in 11 tumor types (LGG (brain lower grade glioma), LUAD, KIRP, KIPAN (pankidney cohort), LIHC, BLCA, SKCM-M (skin cutaneous melanoma-metastasis), MESO, PAAD, LAML (acute myeloid leukemia), and ALL (acute lymphoblastic leukemia)) (Figure 4(a)). Conversely, in STES (stomach and esophageal carcinoma) and STAD tumors, PAQR4 was a protective factor. PAQR4 expression also affected patient DSS, DFI, and PFI (Figures 4(b)–4(d)).

Additionally, PrognoScan was used to determine the connection between PAQR4 expression and survival in the GEO datasets. The high PAQR4 expression group had worse OS in lung, bladder, and brain cancers (Figure S1). Meanwhile, the PAQR4 high expression group showed worse relapse-free survival in lung and breast cancers. The PAQR4 high expression group had a worse DFS for breast cancer. The comparisons were statistically significant; more details are presented in Table 1. These results demonstrate that PAQR4 expression affects the prognosis of patients in multiple cancers.

### 3.3. PAQR4 Genetic Alterations Occur in Different Tumor Tissues

**Figure 5:**
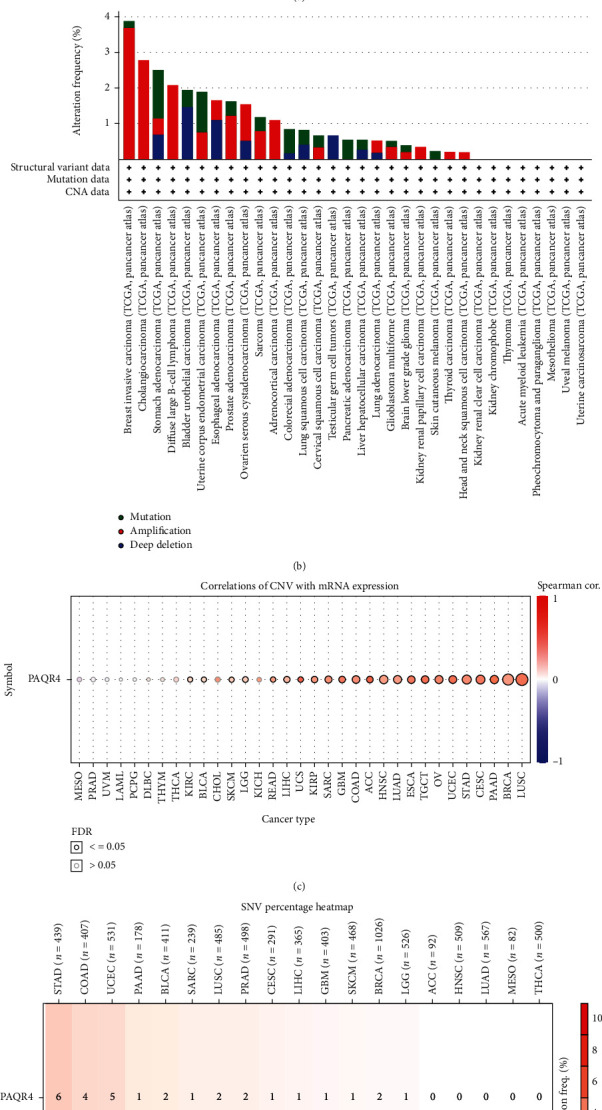
(a, b) PAQR4 genomic alterations in pancancer analyzed by the cBioPortal database. PAQR4 gene alteration occurred in 1.1% of cancer patients. (c) Analysis of the correction between PAQR4 expression and CNV in different tumors in the GSCA website. (d) The frequency of SNV (single nucleotide variants) of PAQR4 in tumors in the GSCA website.

PAQR4 alterations in different cancers were analyzed using cBioPortal. PAQR4 gene alterations occurred in 1.1% of the patients with cancer (Figure 5(a)). Amplification was the main type of PAQR4 alteration. Missense mutations in PAQR4 gene also occurred in a patient subset. A summary of pancancer PAQR4 mutations showed the highest frequency of PAQR4 amplifications in invasive breast carcinoma (Figure 5(b)). The most frequent alteration in cholangiocarcinoma was amplification, and amplification occurred in diffuse large B-cell lymphoma and adrenocortical carcinoma (Figure 5(b)).

Using the GSCA website, we further examined the connection between PAQR4 expression and CNV in other cancers. PAQR4 and CNV significantly correlated in 23 tumors (Figure 5(c)). Concurrently, we analyzed the frequency of SNV (single nucleotide variants) of PAQR4 in tumors. SNV frequency was highest in STAD (Figure 5(d)). These results demonstrated that PAQR4 alterations occur in different tumor types and may regulate tumorigenesis and proliferation.

### 3.4. Pancancer Analysis of PAQR4 Promoter Methylation

**Figure 6:**
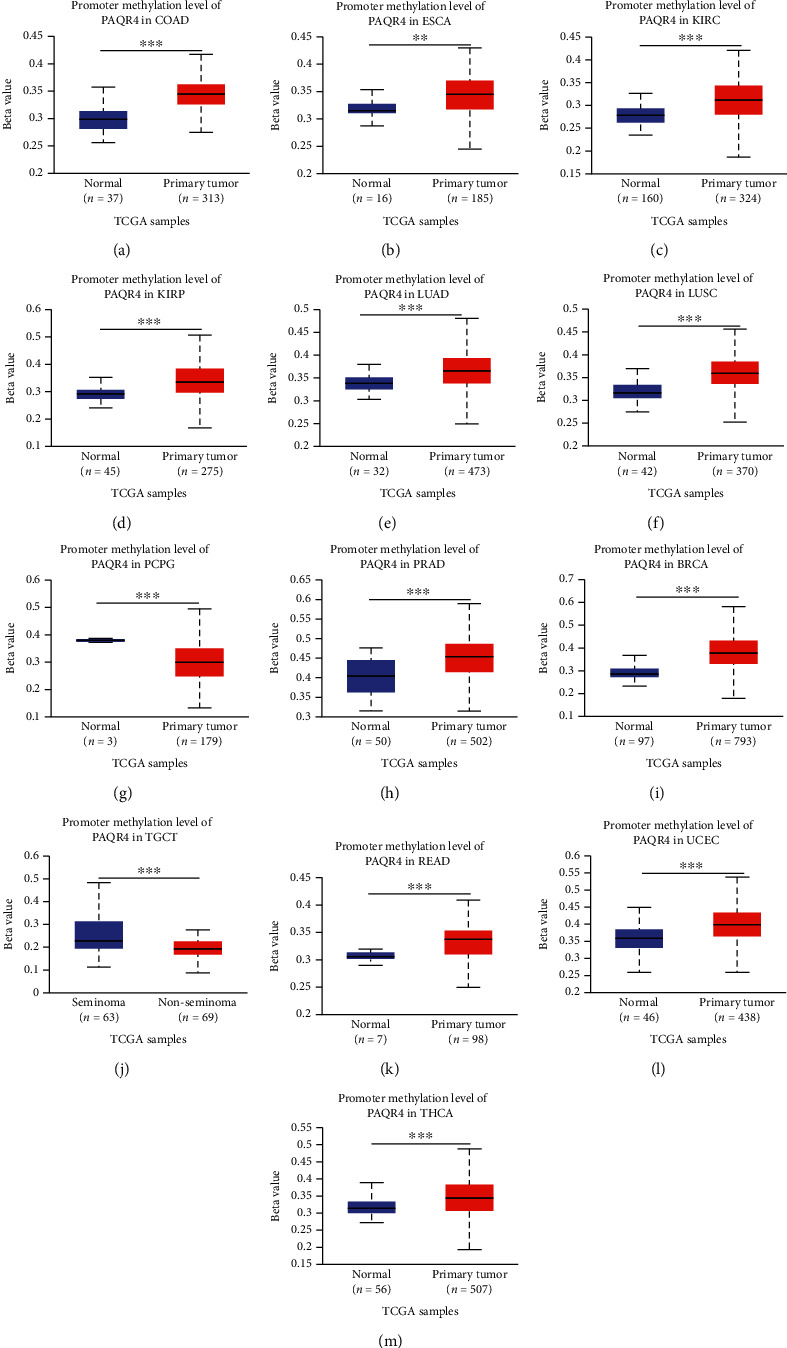
The DNA promoter methylation levels between normal and cancer tissues in the UALCAN database.

DNA methylation is closely associated with tumor development, metastasis, and progression [21]. Epigenetic methylation controls tumor proliferation and metastasis [22–24] and can be used to monitor the effectiveness of tumor risk interventions [23]. Moreover, DNA methyltransferases are important research targets for epigenetic tumor drug therapy [25]. The UALCAN website was used to analyze PAQR4 promoter methylation levels across tumors. PAQR4 promoter methylation levels in COAD, ESCA, KIRC, KIRP, LUAD, LUSC (lung squamous cell carcinoma), PRAD (prostate adenocarcinoma), BRCA, READ, THCA (thyroid carcinoma), and UCEC were substantially higher compared to those in normal tissues, while the promoter methylation levels in PCPG (pheochromocytoma and paraganglioma) and TGCT were significantly lower than those in normal tissues (Figure 6), suggesting that there are differences in PAQR4 promoter methylation levels in different tumors.

Subsequently, the Sangerbox was used to analyze the correlation of PAQR4 with three RNA modification (m1A, m5C, and m6A) marker genes (Figure S2). Most of these genes' expression and PAQR4 have a positive correlation. Based on this data, we speculate that PAQR4 regulates tumorigenesis at the epigenetic level and further influences tumor progression.

### 3.5. PAQR4 Expression Is Correlated with Pancancer Immune Subtypes

**Figure 7:**
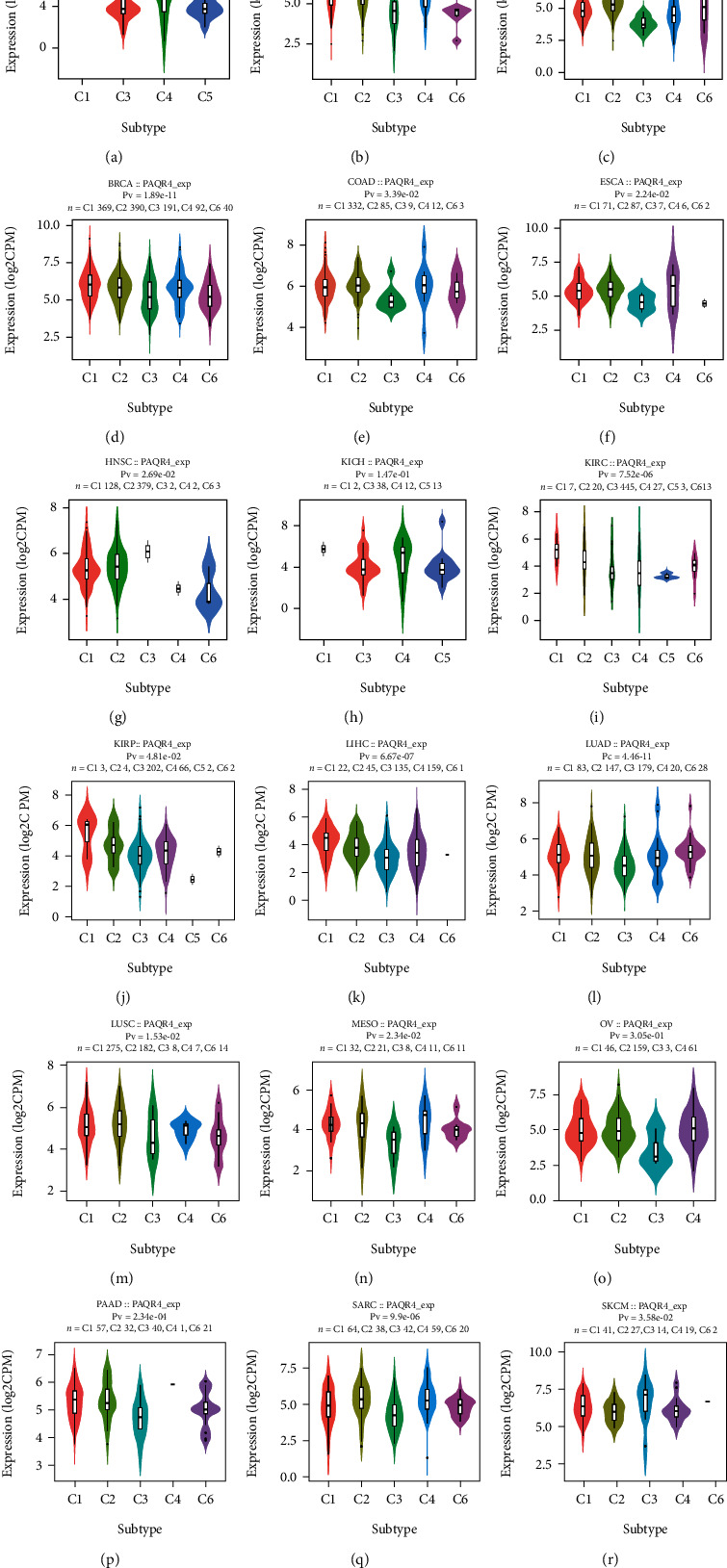
(a–r) The relationship between PAQR4 expression and six immune subtypes across pancancer, including C1-C6 subtypes. In different subtypes, PAQR4 expression is different.

Immune subtypes within tumors suggest a cancer's immune status. These subtypes represent the tumor microenvironment characteristics of different tumor types [26] and guide tumor immunotherapy. Immune subtypes include six types. C1 (wound healing) type has increased angiogenic gene expression, high proliferation rate, and TH2 cell bias towards adaptive immune infiltration. The strongest M1/M2 macrophage polarization is found in C2 (IFN-*γ*-dominant), and it shows a high proliferation rate. C3 (inflammatory) has increased Th17 and Th1 gene expression. C4 (lymphocyte-depleted) showed more macrophage characteristics: Th1 inhibition and high M2 reactivity. C5 (immunologically quiet) showed the minimum lymphocyte count and the highest macrophage response. C6 (TGF-*β*-dominant) cells showed the highest TGF-*β* signal and high lymphocyte infiltration [27]. Using the TISIDB immunological website, we examined the relationship between PAQR4 and immune subtypes in several cancers. (Figure 7). We found different PAQR4 expression levels of different immune subtypes within the same tumor. For example, in BLCA, PAQR4 expression was higher in C2 and C6 and lower in C3. In KIRP, PAQR4 expression was significantly higher in C1 than in other types. The findings above show that PAQR4 is related to immune subtypes and may regulate the tumor-immune environment.

### 3.6. Correlation of PAQR4 Expression Level with TMB, MSI, NEO, Purity, and ICP Genes in Tumors

ICP genes are 60 genes representing the two immune checkpoint pathways (inhibitory (24 genes) and stimulatory (36 genes)) [27]. TMB, MSI, and NEO are considered important biomarkers of the tumor microenvironment [28, 29]. Cell-intrinsic factors and metabolites in the tumor microenvironment affect the metabolism and behavior of cancer cells, which is a key link in cancer development, progression, metastasis, and drug resistance [30]. The tumor microenvironment directly affects the efficacy of therapies including chemotherapy and immunotherapy. A significant relationship between TMB and the objective remission rate of anti-PD-1 or anti-PD-L1 therapy has been reported for several cancers [31], and MSI, a biomarker of PD-1 blockade, is strongly associated with tumor diagnosis and immunotherapy efficacy.

**Figure 8:**
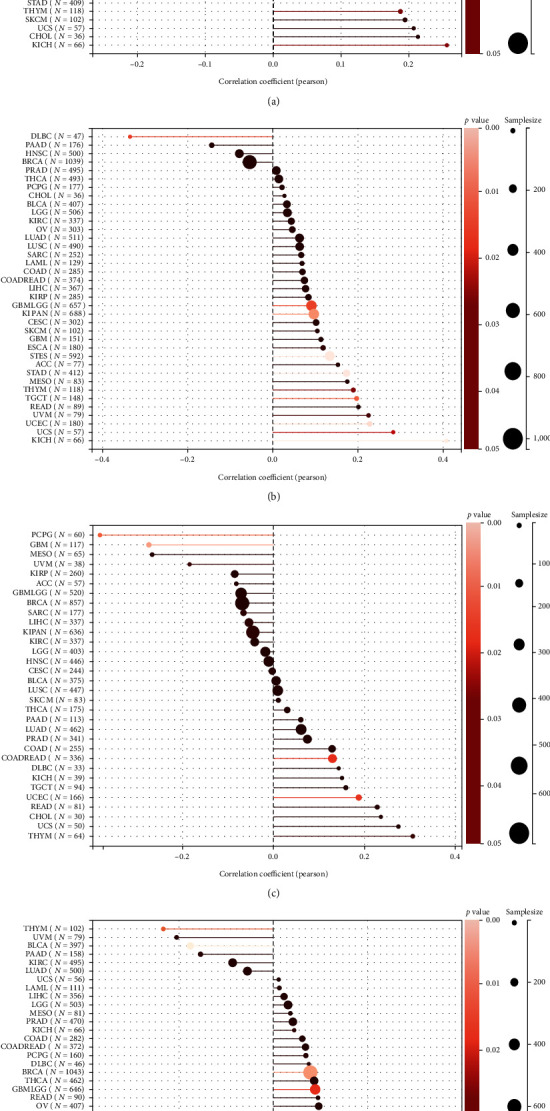
The relationship between PAQR4 expression and (a) TMB, (b) MSI, (c) NEO, (d) purity. (^∗^*P* < 0.05).

To explore the immune mechanism of PAQR4 in the tumor microenvironment, the Pearson correlation coefficient was calculated between PAQR4 expression level and TMB, MSI, NEO, and purity in pancancer using the Sangerbox. PAQR4 was significantly positively correlated with TMB in seven tumors (LUAD, STES, KIPAN, STAD, UCEC, THYM, and KICH) (Figure 8(a)). PAQR4 was significantly correlated with MSI, with a positive correlation in ten tumors (GBMLGG (glioma), STES, KIPAN, STAD, UCEC, THYM, TGCT, UVM, UCS, and KICH) and a negative correlation in one tumor (DLBC) (Figure 8(b)). PAQR4 was significantly correlated with NEO, including positive correlations in three tumors (COADREAD (colon adenocarcinoma/rectum adenocarcinoma esophageal carcinoma), UCEC, and THYM) and negative correlations in two tumors (GBM and PCPG) (Figure 8(c)).

Additionally, the relationship between PAQR4 expression and tumor purity was examined. There was a significant correlation in 18 tumors, a positive correlation in GBM, GBMLGG, CESC, BRCA, ESCA, STES, SARC, KIRP, etc., and a positive correlation in THYM and BLCA (Figure 8(d)). Next, we analyzed the correlation between ICP genes and PAQR4 and found that PAQR4 was significantly positively correlated with CD276, VEGFA, and HMGB1 in most tumors and was also positively correlated with most ICP genes in LIHC, OV, BLCA, and KIRC (Figure S3). Thus, PAQR4 is associated with various biomarkers of the tumor microenvironment and has the potential to be a tumor immunotherapy target.

### 3.7. Correlation Analysis of PAQR4 Expression Level with ESTIMATE in the Tumor Microenvironment

Researchers can obtain ratings for tumor purity, the number of stromal cells present, and the degree of immune cell infiltration in tumor tissues utilizing the ESTIMATE. We looked further at the relationship between stromal and immunological scores as well as ESTIMATE scores and PAQR4 expression.

**Figure 9:**
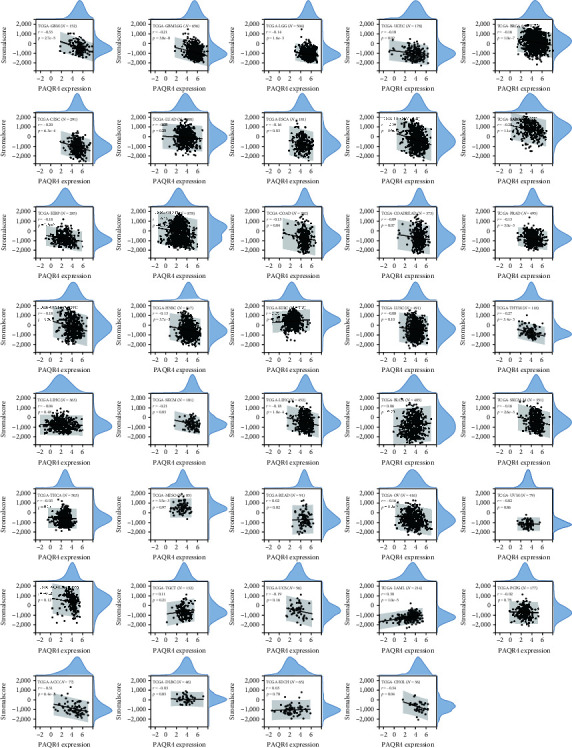
The correlation between PAQR4 expression and stromal score. 22 of 23 tumors are negative correlations.

Among the 39 tumor types, we observed significant correlations between PAQR4 expression and stromal scores in 23 tumor types, including 22 negative correlations (Figure 9). PAQR4 was significantly correlated with the immune scores for 19 cancer types, including 17 negative correlations (Figure S4). PAQR4 expression level was significantly correlated with the ESTIMATE scores among 23 cancers, including 20 negative correlations (Figure S5). Next, we investigated the correlation between PAQR4 expression and the degree of infiltration of six immune cells: B cells, CD8 + T cells, CD4 + T cells, neutrophils, dendritic cells, and macrophages. The degree of infiltration of different immune cells was significantly correlated with PAQR4 expression in BLCA, BRCA, CESC, HNSC, KIRC, LIHC, LUSC, STAD, THYM, KICH, and MESO (Figure S6). Through the above series of immunological studies, PAQR4 has been shown to influence the infiltration of different immune cells into tumors to regulate the immune microenvironment.

### 3.8. Correlation of PAQR4 Expression with Drug Sensitivity

**Figure 10:**
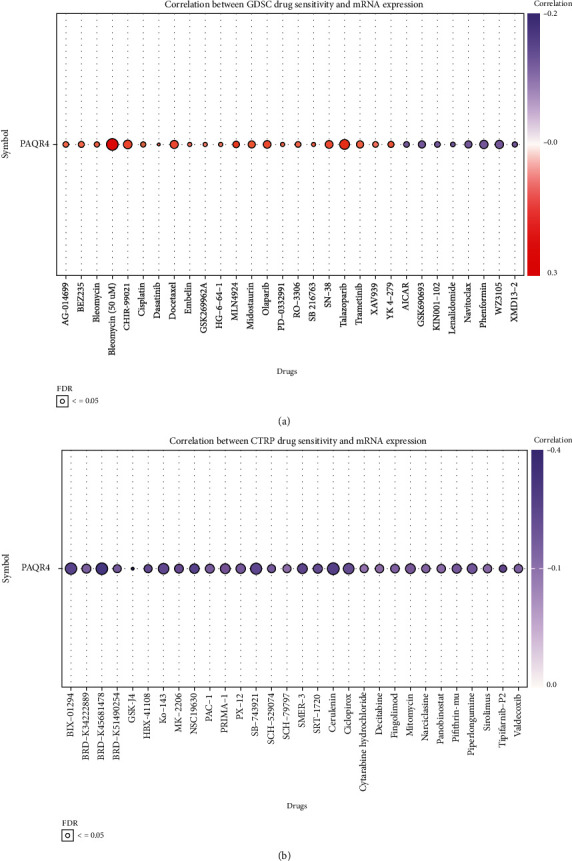
The correlation between the drug sensitivity and mRNA expression in (a) GDSC database and (b) CTRP database.

**Figure 11:**
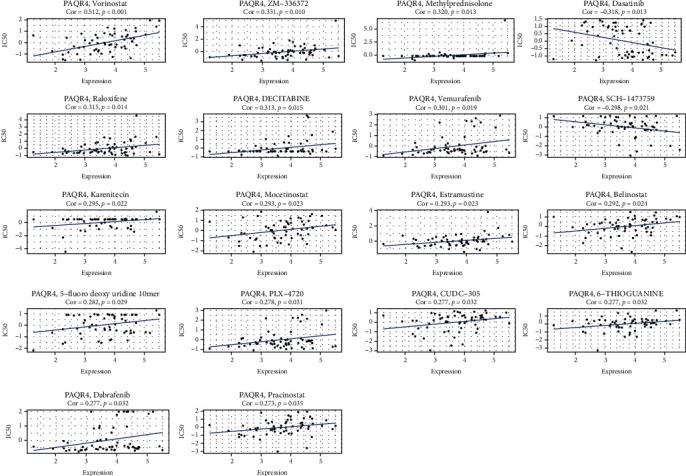
The correlation analysis of PAQR4 expression and drug IC50. The 18 drugs that most significantly correlated with PAQR4 expression.

Chemotherapeutic agents remain the primary choice for first-line treatment of many tumors [32]. The development of tumor drug resistance may be associated with gene dysregulation [33]. Consequently, the efficacy of many therapies is greatly compromised. Thus, we examined the connection between medication sensitivity and PAQR4 expression using the GSCA website to mine the GDSC and CTRP databases. PAQR4 expression was significantly associated with multidrug sensitivity. The GDSC database results showed significant positive correlations with sensitivity to bleomycin, talazoparib, docetaxel, midostaurin, and trametinib (Figure 10(a)). The CTRP database showed that PAQR4 was significantly negatively correlated with the sensitivity to cytarabine hydrochloride, decitabine, fingolimod, mitomycin, panobinostat, and sirolimus (Figure 10(b)). Using the CellMiner database to analyze PAQR4 and drug IC50s, 18 significant relationships were uncovered showing positive PAQR4 association with the IC50 of most drugs, including vorinostat, methylprednisolone, decitabine, vemurafenib, karenitecin, 5-Flu, and dabrafenib (Figure 11). These drugs are used in chemotherapy, endocrine therapy, and targeted therapy for tumors. Some of these drugs are the most recently approved novel drugs for oncology regimens, and some are well-established for oncologic chemotherapy. These results indicate that the development of drug resistance in different tumors may be related to the abnormal expression of PAQR4 and provides an alternative diagnostic test for precision therapy.

## 4. Discussion

Cancer is a major disease that threatens billions of lives worldwide [34]. It is difficult to diagnose in early stages, and late stage metastasis makes it the number one killer of human beings [35]. Research on cancer has gradually progressed from the initial pathological diagnosis to the genetic level [36]. However, we find that the tumor is a multilevel regulated genetic disease. The heterogeneity of tumors and the complex immune microenvironment leave the pathogenesis of tumors in an unclear position. New therapeutic tools for tumors have also been discovered, although chemotherapy remains the first-line treatment for most tumors [37]. However, there are many problems in clinical practice including chemotherapeutic drug side effects. Granulocytopenia and chemotherapeutic resistance are observed in tumors with distant metastases [38]. These side effects expose patients to risks that are disproportionate to the benefits. With the identification of molecular targets and their cellular functions, targeted therapies based on antibodies and small-molecule inhibitors have begun to be applied [39]. With the focus on tumor immunity research, the regulation of tumors by the immune system provides new hope for tumor treatment. More targets and mutation sites have been identified, and drugs that are targeting genes such as EGFR and HER2 have been shown to have definite antitumor effects in breast and lung cancers [40]. However, the development of small-molecule drugs is not perfect, and there are still many challenges in the regulation of tumor metabolism and inhibitors of tumor gene mutations [41]. Many patients remain insensitive to the current small-molecule target drugs, and these small-molecule-targeted drugs based on modulation of the innate immune system, its side effects, and antitumor effects are still unclear [42]. Meanwhile, numerous clinical trials demonstrated that immunotherapy based on PD-1 and PD-L1 inhibitors significantly prolongs patient survival [43]. Owing to the difficulty of early tumor diagnosis and the complex immune environment, the current therapeutic tools to treat tumors are far from sufficient, and new targets are urgently needed to guide precision medicine [44].

To discover more valuable targets and explore commonalities in tumor development, we used bioinformatic techniques to analyze individual gene expression differences across cancers at the transcriptome level. In the analysis of PAQR4 across pancancer, based on data from TCGA and GTEx databases, we found that PAQR4 expression levels were significantly upregulated in up to thirty tumors. Its prevalent high levels are difficult to ignore. And its ability to promote cell proliferation, which drives cellular carcinogenesis and unlimited proliferation, has been well established in existing studies on individual tumors. The oncogenic role of PAQR4 in LIHC, NSCLC, and BRCA has been demonstrated in experimental studies. Further, we explored the prognostic value of PAQR4, and prognostic indicators, such as OS, DSS, DFI, and PFI, are closely correlated with PAQR4 expression. High PAQR4 expression is a risk factor for most tumors, and its higher expression is associated with shorter survival, which significantly affects patient prognosis. These results indicated that PAQR4 promotes oncogenesis and has the potential to be a pancancer prognostic marker. Next, PAQR4 was investigated from a genetic and epigenetic perspective. We found significantly higher levels of PAQR4 promoter methylation in most of tumors, and PAQR4 was significantly associated with most of the RNA modification marker genes. These epigenetic alterations may cause dysregulation of PAQR4 in many cancers and can act as risk markers for tumors [45].

The six immune subtypes characterize different immune states within the tumor and are closely related to tumor prognosis, genetics, and immune regulation. Our analysis showed significant differences in PAQR4 expression among different immune subtypes. PAQR4 is involved in immune regulation of the tumor environment, causing tumors to develop toward different immune subtypes. PAQR4 may regulate the distribution as well as the ratio of different cell subpopulations in the immune microenvironment and contribute to changes in tumor purity. Next, we examined the correlation between TMB, MSI, and NEO in the tumor microenvironment. The total number of somatic genetic coding mistakes, base substitutions, insertions, or deletions found per million bases is known as TMB [46]. TMB is an indicator of the efficacy of anti-PD1/PDL1 therapy in tumors, and persistently high TMB improves efficacy of immune checkpoint blockade therapy [29, 47]. Similarly, MSI significantly affects tumor prognosis [48]. TMB in seven tumor types significantly positively correlated with PAQR4 expression, and MSI of 10 types of tumors was significantly correlated with PAQR4 expression, including a significant negative correlation in DLBC. Thus, PAQR4 influences tumorigenesis by participating in multiple gene alterations and has the potential to be an important marker for cancer immunotherapy and prognosis. The tumor-specific antigen NEO, which is produced by nonsynonymous mutations, is a highly desirable target for tumor immunotherapy. Numerous tumor cells express NEO, which has a potent immunogenicity and a diverse range of tumor types [49]. As a new approach to tumor immunotherapy, vaccinations produced against NEO have been used in clinical studies for several solid tumors [50], and the NEO of five types of tumors was significantly associated with PAQR4 in this study.. All above revealed that PAQR4 plays a complex role in tumors and is a promising target.

In addition to tumor cells, there are nontumor cells such as immune cells, stromal cells, and mesenchymal cells in tumor tissues, and these different types of cells together constitute a complex tumor microenvironment [51], which often limits or poorly differentiates vasculature, creating inefficiencies in nutrient and oxygen delivery as well as waste removal [52]. Metastasis depends on bidirectional interactions between cancer cells and their environment [53]. The tumor microenvironment is closely related to patient clinical characteristics, genomic expression, and biological properties [54]. Our research provided a significant correlation between PAQR4 expression and the tumors purity in 18 cancers, especially in THYM and BLCA, where higher PAQR4 was associated with lower tumor purity. In the remaining 16 tumors, the higher the PAQR4 expression, the higher the tumor purity. We also correlated PAQR4 expression with the ESTIMATE score in the tumor microenvironment and found that stromal, immunological, and ESTIMATE scores were negatively correlated with PAQR4 expression in more than 3/4 of tumors, which was consistent with our previous analysis. Based on these immunological studies, PAQR4 expression can significantly affect the composition of different immune cells in the tumor microenvironment and participate in different pathways to modulate the immune response of the tumor, which may be associated with poor prognosis in the high PAQR4 group.

Previous investigations revealed that PAQR4 is involved in the regulation of chemotherapy resistance in NSLC [12], and our analysis of drug databases revealed that PAQR4 expression is significantly associated with sensitivity to multiple chemotherapeutics. These drugs constitute the treatment protocols for different oncology patients. We will further explore the specific mechanisms underlying PAQR4 involvement in chemotherapy resistance in different cancers, which is important for guiding personalized clinical drug decisions.

While we extensively analyzed PAQR4 and cross-validated with multiple databases, many limitations remained in this study. First, biases in sequencing data from different databases might have led to systematic errors. Second, further cellular or animal models are required to validate the potential PAQR4 functions. Although we concluded that PAQR4 can influence tumor progression through the immune environment, the exact regulatory mechanisms remain unclear, and there remain multiple avenues for PAQR4 research. In the future, we will prospectively investigate PAQR4's function and attempt to develop and test novel antitumor immunotherapeutic agents targeting PAQR4 to enable more precise treatment of malignant tumor patients.

## 5. Conclusion

Transcriptomic data from different sources were analyzed at the tissue and cellular levels to understand the differences in PAQR4 expression between tumor and normal groups. We found that PAQR4 was significantly upregulated in tumors and affected patient survival. We further investigated the correlation between PAQR4 and infiltration of different lymphocyte subpopulations and the IC50 of different drugs. These findings suggest that PAQR4 is a potential immunomodulator with important implications for tumor therapy. The above pancancer studies of PAQR4 suggest that PAQR4 is a potential target for tumor diagnosis and immunotherapy.

## Figures and Tables

**Table 1:** The relationship between PAQR4 gene expression and the prognosis of different cancers in PrognoScan.

	Dataset	Cancer type	ENDPOINT	Probe ID	Corrected *P* value	In (HR high/HR low)	COX *P* value	In (HR)	95% CI	Reference
A	GSE4922	Breast cancer	Disease-free survival	212858_at	0.001079	0.96	0.002088	0.57	1.76 (1.23-2.52)	PMID: 17079448
B	GSE17710	Lung cancer	Overall survival	27382	0.001399	1.42	0.011716	0.56	1.75 (1.13-2.69)	PMID: 20643781
C	GSE16581	Brain cancer	Overall survival	212858_at	0.023357	2.08	0.143075	2.67	14.46 (0.40-516.58)	PMID: 20015288
D	GSE13507	Bladder cancer	Overall survival	ILMN_1660793	0.002041	0.97	0.000628	0.43	1.54 (1.20-1.98)	PMID: 20059769
E	GSE31210	Lung cancer	Relapse-free survival	212858_at	0.006116	1.02	0.008767	0.88	2.40 (1.25-4.63)	PMID: 22080568
F	GSE1456	Breast cancer	Relapse-free survival	212858_at	0.015712	1.06	0.009348	0.83	2.30 (1.23-4.32)	PMID: 16813654
G	GSE30929	Soft tissue cancer	Distant recurrence free survival	212858_at	0.017279	0.99	0.00357	1.49	4.46 (1.63-12.18)	PMID: 21335544
H	GSE3494	Breast cancer	Disease-specific survival	212858_at	0.035271	1.10	0.003606	0.68	1.98 (1.25-3.12)	PMID: 16141321
I	GSE13507	Bladder cancer	Disease-specific survival	ILMN_1660793	0.011424	1.27	0.000679	0.63	1.88 (1.30-2.69)	PMID: 20059769

## Data Availability

The datasets generated and analyzed during the current study are available in the TCGA (https://www.cancer.gov/tcga), GTEx (http://commonfund.nih.gov/GTEx/), and GEO (https://www.ncbi.nlm.nih.gov/geo/).

## Supplementary Materials

Supplementary Materials A total of six supplementary images and one supplementary table are included in this study, as described in the article. Figure S1: (a–i) survival analyses of the PAQR4 in human cancers in the PrognoScan website. Figure S2: the correlation of PAQR4 with three RNA modification (m1A, m5C, and m6A) marker genes, including writers, readers, and erasers through the Sangerbox web. Figure S3: the correlation between PAQR4 expression and ICP genes. PAQR4 was significantly positively correlated with CD276, VEGFA, and HMGB1 in most tumors and was also positively correlated with most ICP genes in LIHC, OV, BLCA, and KIRC. Figure S4: the correlation between PAQR4 expression and immune scores. PAQR4 was significantly correlated with the immune scores for 19 cancer types, including 17 negative correlations. Figure S5: the correlation between PAQR4 expression and ESTIMATE score. PAQR4 expression was significantly correlated with the ESTIMATE scores in 23 cancers. Figure S6: the correlation between PAQR4 expression and the infiltration degree of six immune cells, including B cells, CD8 + Tcells, CD4 + T cells, neutrophils, dendritic cells, and macrophages in BLCA, BRCA, CESC, HNSC, KIRC, LIHC, LUSC, STAD, THYM, KICH, and MESO.
