# Supplementary figures and images for: Pancancer Analysis of the Prognostic and Immunotherapeutic Value of Progestin and AdipoQ Receptor 4
Source: Comput Math Methods Med. 2022 Dec 17;2022:2528164. doi: 10.1155/2022/2528164 (PMC9789910; doi:10.1155/2022/2528164)

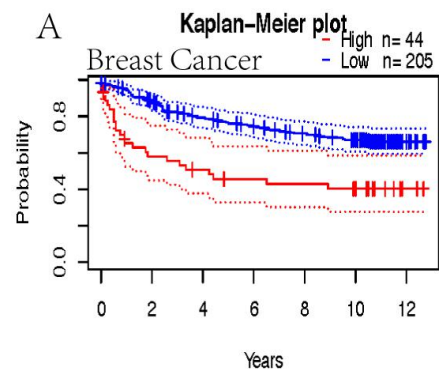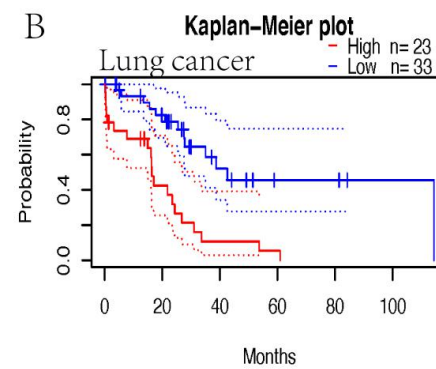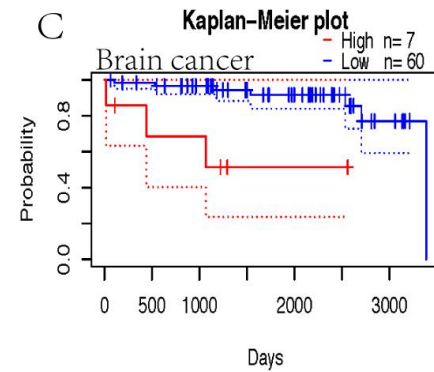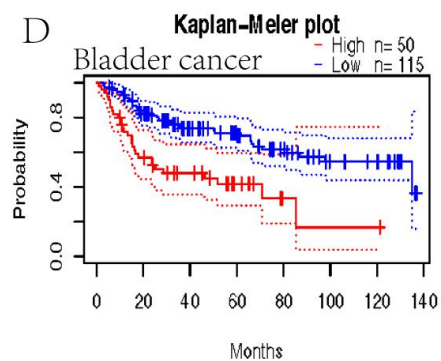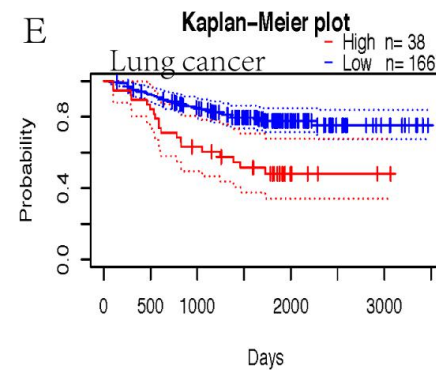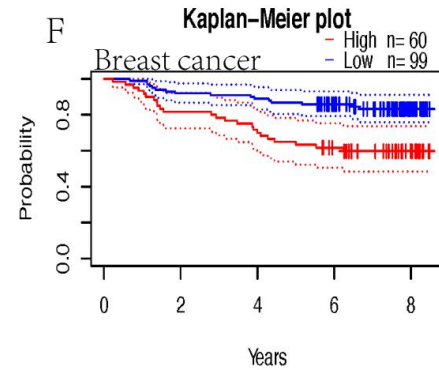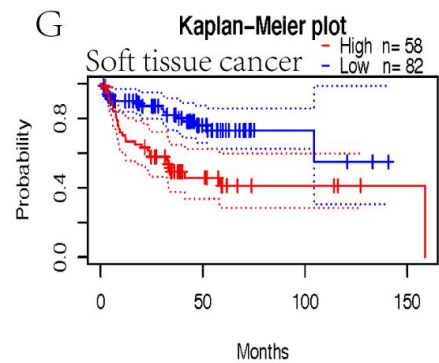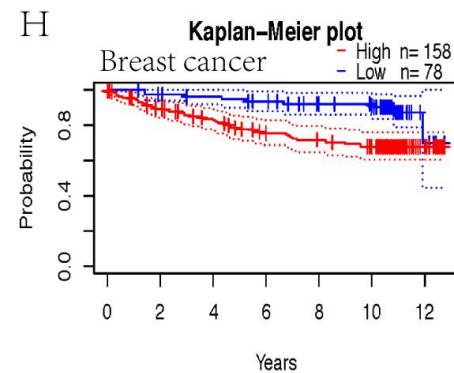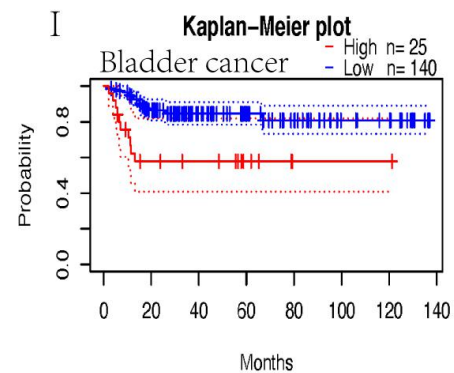

Supplement: Supplementary Materials — A total of six supplementary images and one supplementary table are included in this study, as described in the article. Figure S1: (a–i) survival analyses of the PAQR4 in human cancers in the PrognoScan website. Figure S2: the correlation of PAQR4 with three RNA modification (m1A, m5C, and m6A) marker genes, including writers, readers, and erasers through the Sangerbox web. Figure S3: the correlation between PAQR4 expression and ICP genes. PAQR4 was significantly positively correlated with CD276, VEGFA, and HMGB1 in most tumors and was also positively correlated with most ICP genes in LIHC, OV, BLCA, and KIRC. Figure S4: the correlation between PAQR4 expression and immune scores. PAQR4 was significantly correlated with the immune scores for 19 cancer types, including 17 negative correlations. Figure S5: the correlation between PAQR4 expression and ESTIMATE score. PAQR4 expression was significantly correlated with the ESTIMATE scores in 23 cancers. Figure S6: the correlation between PAQR4 expression and the infiltration degree of six immune cells, including B cells, CD8 + Tcells, CD4 + T cells, neutrophils, dendritic cells, and macrophages in BLCA, BRCA, CESC, HNSC, KIRC, LIHC, LUSC, STAD, THYM, KICH, and MESO. [file 2528164.f1.zip › Figure S1 (1).pdf]

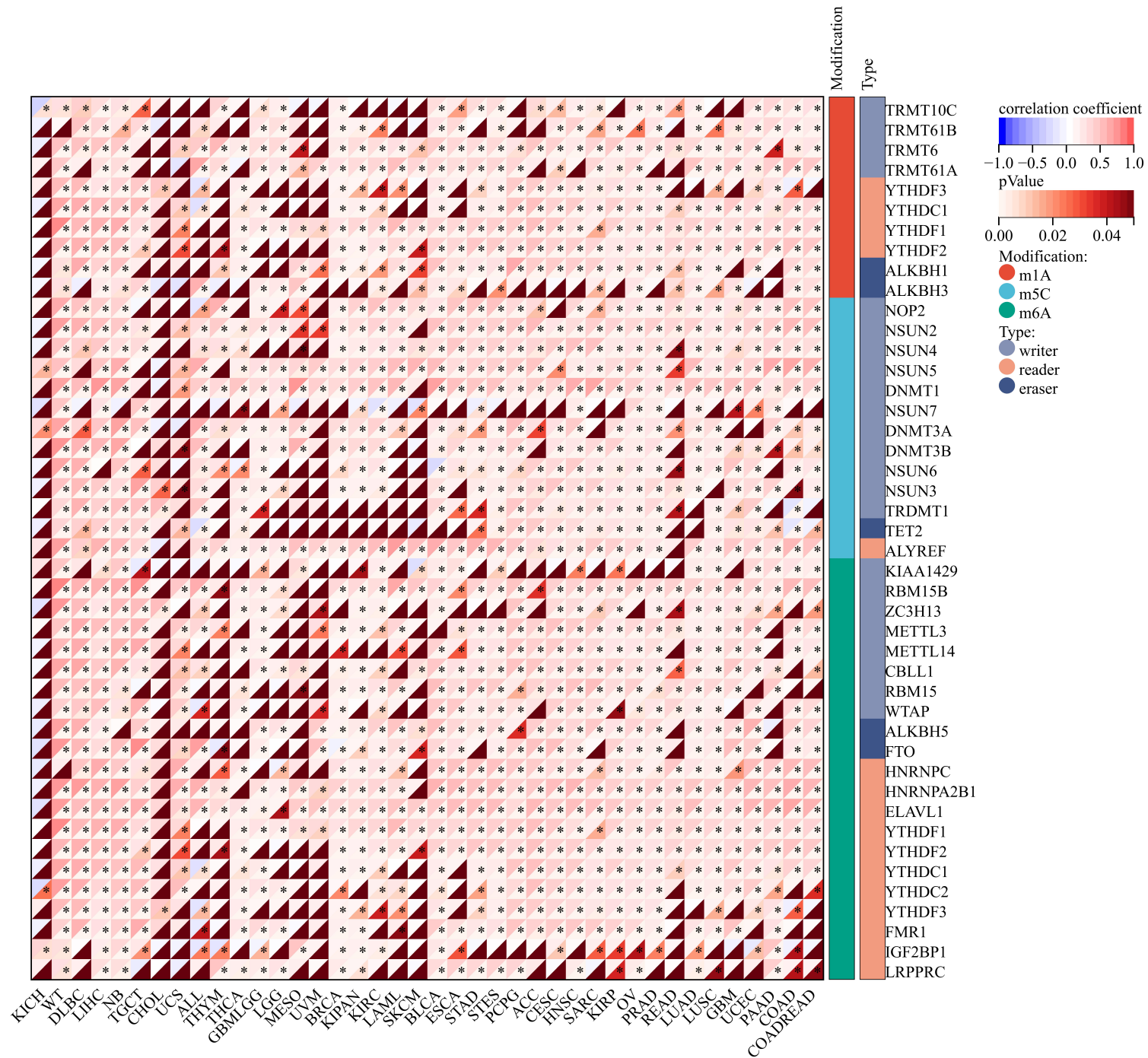

Supplement: Supplementary Materials — A total of six supplementary images and one supplementary table are included in this study, as described in the article. Figure S1: (a–i) survival analyses of the PAQR4 in human cancers in the PrognoScan website. Figure S2: the correlation of PAQR4 with three RNA modification (m1A, m5C, and m6A) marker genes, including writers, readers, and erasers through the Sangerbox web. Figure S3: the correlation between PAQR4 expression and ICP genes. PAQR4 was significantly positively correlated with CD276, VEGFA, and HMGB1 in most tumors and was also positively correlated with most ICP genes in LIHC, OV, BLCA, and KIRC. Figure S4: the correlation between PAQR4 expression and immune scores. PAQR4 was significantly correlated with the immune scores for 19 cancer types, including 17 negative correlations. Figure S5: the correlation between PAQR4 expression and ESTIMATE score. PAQR4 expression was significantly correlated with the ESTIMATE scores in 23 cancers. Figure S6: the correlation between PAQR4 expression and the infiltration degree of six immune cells, including B cells, CD8 + Tcells, CD4 + T cells, neutrophils, dendritic cells, and macrophages in BLCA, BRCA, CESC, HNSC, KIRC, LIHC, LUSC, STAD, THYM, KICH, and MESO. [file 2528164.f1.zip › Figure S2 (1).pdf]

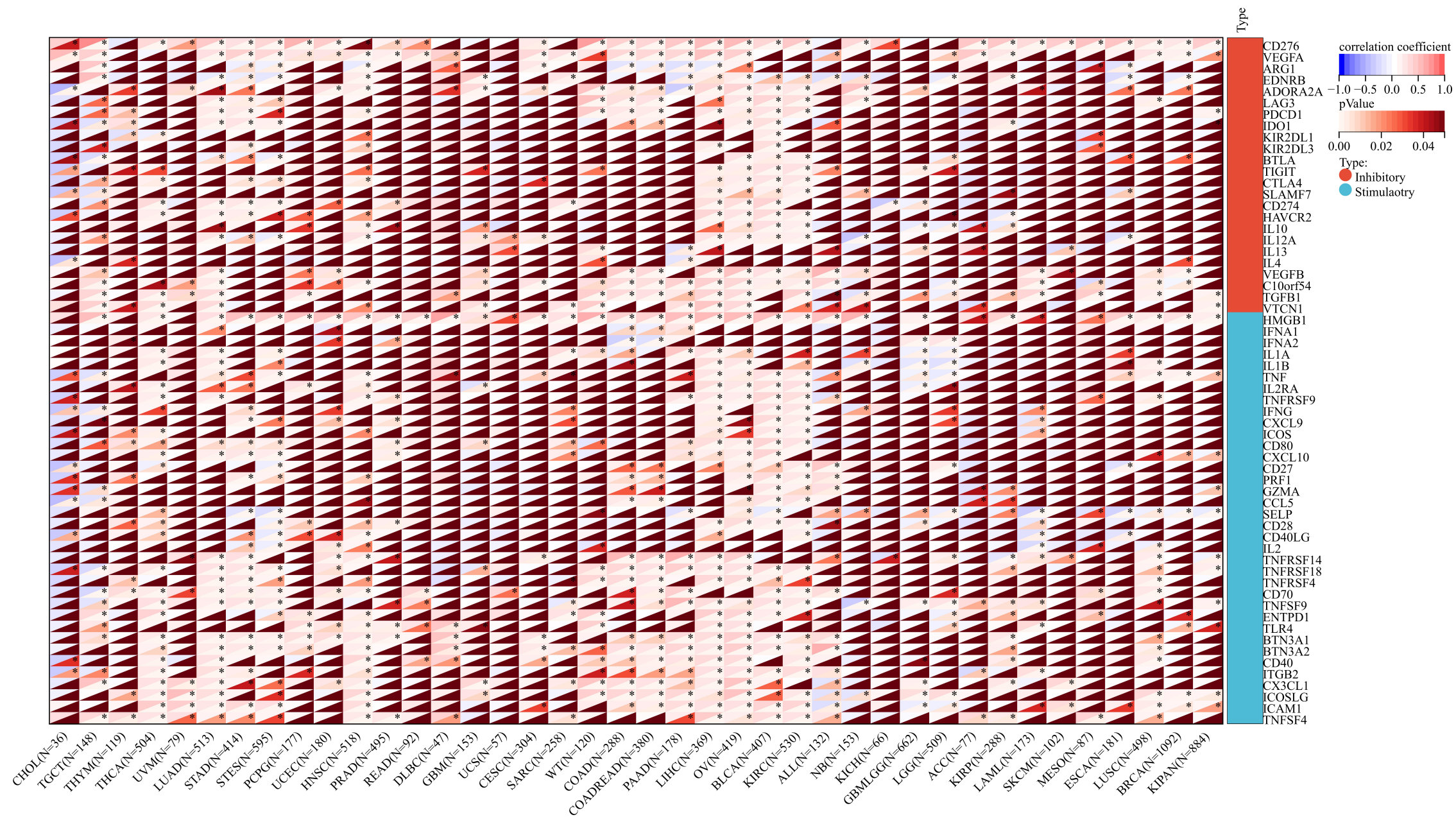

Supplement: Supplementary Materials — A total of six supplementary images and one supplementary table are included in this study, as described in the article. Figure S1: (a–i) survival analyses of the PAQR4 in human cancers in the PrognoScan website. Figure S2: the correlation of PAQR4 with three RNA modification (m1A, m5C, and m6A) marker genes, including writers, readers, and erasers through the Sangerbox web. Figure S3: the correlation between PAQR4 expression and ICP genes. PAQR4 was significantly positively correlated with CD276, VEGFA, and HMGB1 in most tumors and was also positively correlated with most ICP genes in LIHC, OV, BLCA, and KIRC. Figure S4: the correlation between PAQR4 expression and immune scores. PAQR4 was significantly correlated with the immune scores for 19 cancer types, including 17 negative correlations. Figure S5: the correlation between PAQR4 expression and ESTIMATE score. PAQR4 expression was significantly correlated with the ESTIMATE scores in 23 cancers. Figure S6: the correlation between PAQR4 expression and the infiltration degree of six immune cells, including B cells, CD8 + Tcells, CD4 + T cells, neutrophils, dendritic cells, and macrophages in BLCA, BRCA, CESC, HNSC, KIRC, LIHC, LUSC, STAD, THYM, KICH, and MESO. [file 2528164.f1.zip › Figure S3 (1).pdf]

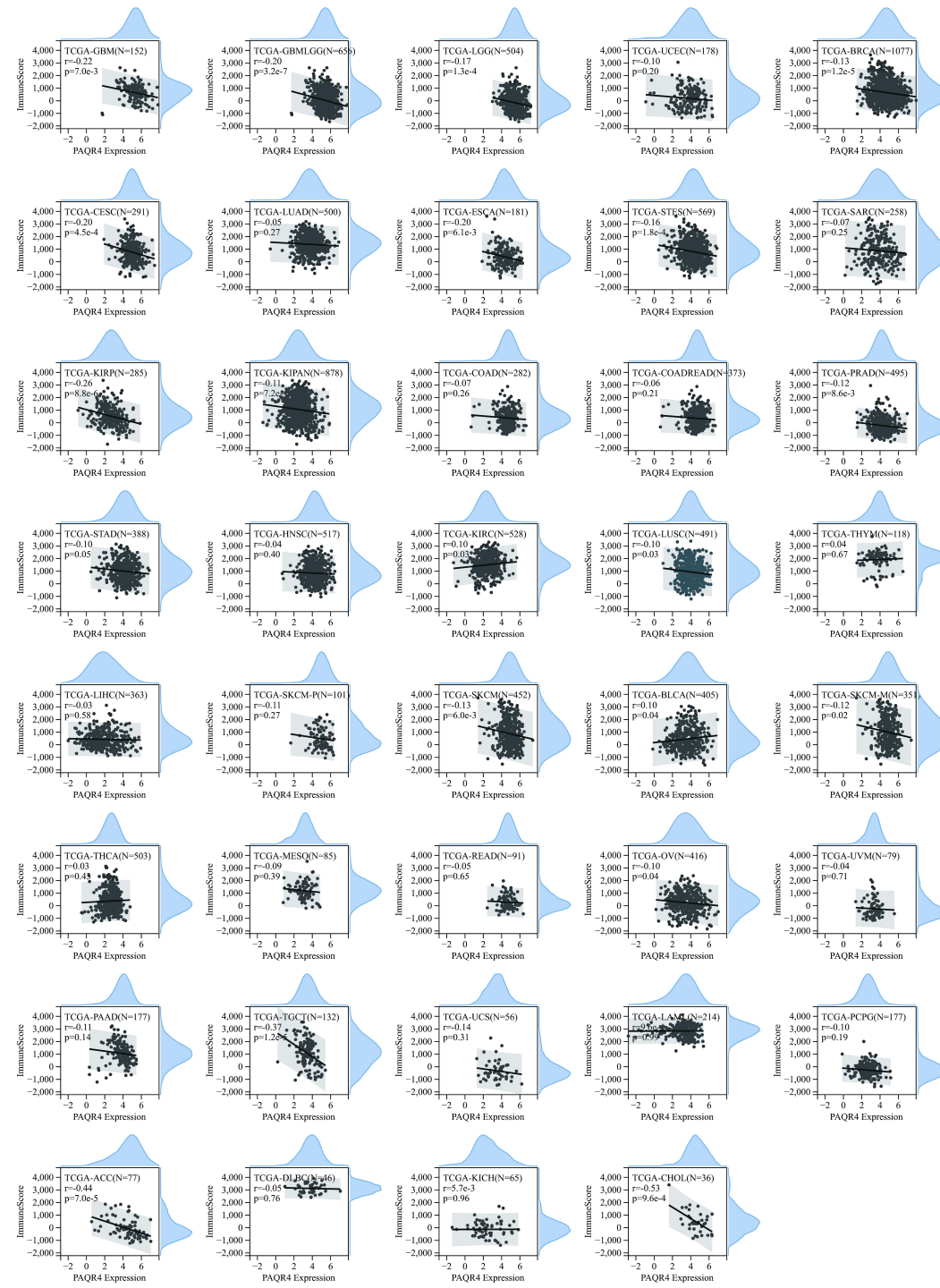

Supplement: Supplementary Materials — A total of six supplementary images and one supplementary table are included in this study, as described in the article. Figure S1: (a–i) survival analyses of the PAQR4 in human cancers in the PrognoScan website. Figure S2: the correlation of PAQR4 with three RNA modification (m1A, m5C, and m6A) marker genes, including writers, readers, and erasers through the Sangerbox web. Figure S3: the correlation between PAQR4 expression and ICP genes. PAQR4 was significantly positively correlated with CD276, VEGFA, and HMGB1 in most tumors and was also positively correlated with most ICP genes in LIHC, OV, BLCA, and KIRC. Figure S4: the correlation between PAQR4 expression and immune scores. PAQR4 was significantly correlated with the immune scores for 19 cancer types, including 17 negative correlations. Figure S5: the correlation between PAQR4 expression and ESTIMATE score. PAQR4 expression was significantly correlated with the ESTIMATE scores in 23 cancers. Figure S6: the correlation between PAQR4 expression and the infiltration degree of six immune cells, including B cells, CD8 + Tcells, CD4 + T cells, neutrophils, dendritic cells, and macrophages in BLCA, BRCA, CESC, HNSC, KIRC, LIHC, LUSC, STAD, THYM, KICH, and MESO. [file 2528164.f1.zip › Figure S4 (1).pdf]

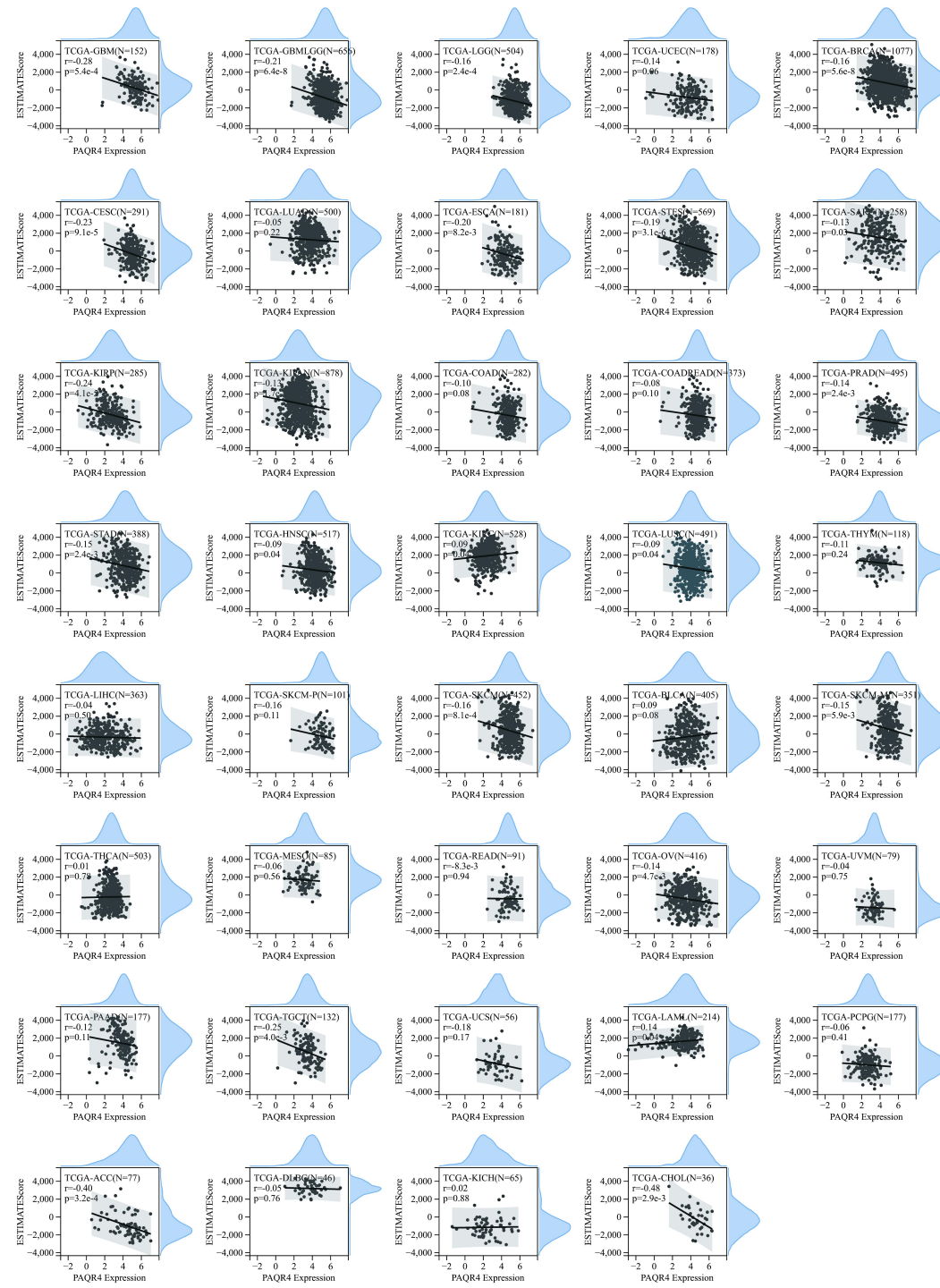

Supplement: Supplementary Materials — A total of six supplementary images and one supplementary table are included in this study, as described in the article. Figure S1: (a–i) survival analyses of the PAQR4 in human cancers in the PrognoScan website. Figure S2: the correlation of PAQR4 with three RNA modification (m1A, m5C, and m6A) marker genes, including writers, readers, and erasers through the Sangerbox web. Figure S3: the correlation between PAQR4 expression and ICP genes. PAQR4 was significantly positively correlated with CD276, VEGFA, and HMGB1 in most tumors and was also positively correlated with most ICP genes in LIHC, OV, BLCA, and KIRC. Figure S4: the correlation between PAQR4 expression and immune scores. PAQR4 was significantly correlated with the immune scores for 19 cancer types, including 17 negative correlations. Figure S5: the correlation between PAQR4 expression and ESTIMATE score. PAQR4 expression was significantly correlated with the ESTIMATE scores in 23 cancers. Figure S6: the correlation between PAQR4 expression and the infiltration degree of six immune cells, including B cells, CD8 + Tcells, CD4 + T cells, neutrophils, dendritic cells, and macrophages in BLCA, BRCA, CESC, HNSC, KIRC, LIHC, LUSC, STAD, THYM, KICH, and MESO. [file 2528164.f1.zip › Figure S5 (1).pdf]

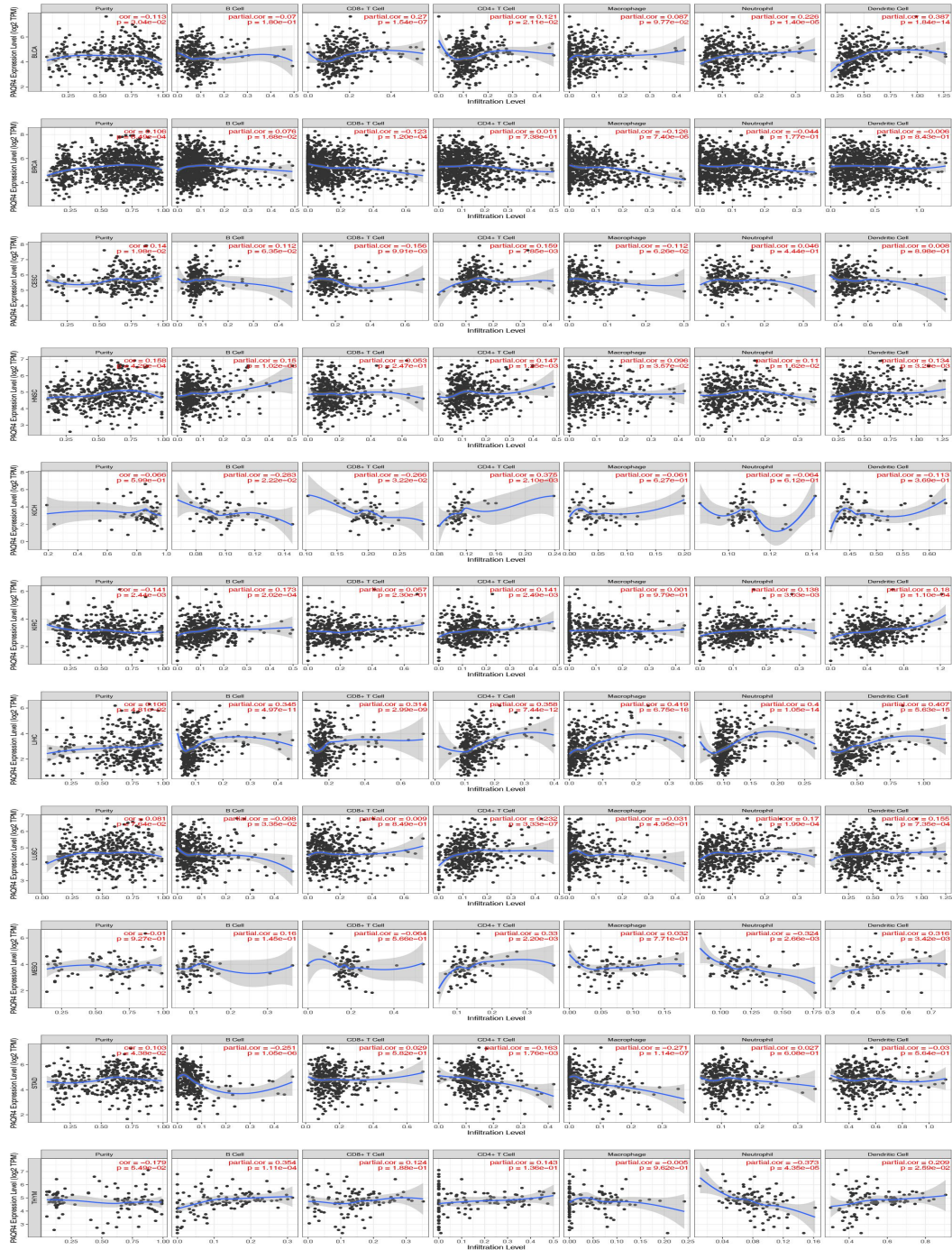

Supplement: Supplementary Materials — A total of six supplementary images and one supplementary table are included in this study, as described in the article. Figure S1: (a–i) survival analyses of the PAQR4 in human cancers in the PrognoScan website. Figure S2: the correlation of PAQR4 with three RNA modification (m1A, m5C, and m6A) marker genes, including writers, readers, and erasers through the Sangerbox web. Figure S3: the correlation between PAQR4 expression and ICP genes. PAQR4 was significantly positively correlated with CD276, VEGFA, and HMGB1 in most tumors and was also positively correlated with most ICP genes in LIHC, OV, BLCA, and KIRC. Figure S4: the correlation between PAQR4 expression and immune scores. PAQR4 was significantly correlated with the immune scores for 19 cancer types, including 17 negative correlations. Figure S5: the correlation between PAQR4 expression and ESTIMATE score. PAQR4 expression was significantly correlated with the ESTIMATE scores in 23 cancers. Figure S6: the correlation between PAQR4 expression and the infiltration degree of six immune cells, including B cells, CD8 + Tcells, CD4 + T cells, neutrophils, dendritic cells, and macrophages in BLCA, BRCA, CESC, HNSC, KIRC, LIHC, LUSC, STAD, THYM, KICH, and MESO. [file 2528164.f1.zip › Figure S6 (1).pdf]
